# Postprandial triglyceride response in normolipidemic, hyperlipidemic and obese subjects – the influence of polydextrose, a non-digestible carbohydrate

**DOI:** 10.1186/s12937-015-0009-0

**Published:** 2015-03-08

**Authors:** Kirsti Tiihonen, Nina Rautonen, Esa Alhoniemi, Markku Ahotupa, Julian Stowell, Tommi Vasankari

**Affiliations:** 1 DuPont Nutrition and Health, Active Nutrition, Sokeritehtaantie 20, FI-02460, Kantvik, Finland; 2 Danisco Health and Nutrition, Kantvik, Finland; 3 Avoltus Oy, Turku, Finland; 4 MCA Research Laboratory, University of Turku, Turku, Finland; 5 DuPont Nutrition and Health, Reigate, UK; 6 UKK Institute for Health Promotion Research, Tampere, Finland

**Keywords:** Postprandial triglycerides, Hyperlipidemia, Hypertriglyceridemia, Obesity, Dietary fiber

## Abstract

**Background:**

Three independent trials were conducted to evaluate postprandial triglyceride (TG) responses in subjects with different lipid metabolism. The effect of polydextrose (PDX), a soluble non-digestible carbohydrate, on postprandial response was also studied using practically relevant, high fat meal interventions.

**Methods:**

A total of 19 normolipidemic (average BMI 24.1 kg/m^2^), 21 overweight/hyperlipidemic (average BMI 29.6 kg/m^2^) and 18 obese/non-diabetic subjects (average BMI 33.6 kg/m^2^) were included in the study. On two separate occasions all subjects ate two high-fat meals (4293 kJ, 36% from fat), one with PDX (either 12.5 g or 15 g) and one without PDX during placebo-controlled, double-blind, crossover and randomized trials. To obtain the triglyceride measurements venous blood samples were taken before the consumption of the test meal and five times afterwards, up to 6 h post-test meal. The triglyceride responses were modeled using a mixed-effects linear model.

**Results:**

The key variables that explain the variation of the postprandial triglyceride response in the different subject groups were: baseline triglyceride concentration, time point, and PDX vs. placebo treatment (p < 0.05). The maximum postprandial TG concentration was more pronounced in hyperlipidemic group compared to normolipidemic (p < 0.001) or obese groups (p < 0.01). The modeled TG response analysis showed that irrespective of the study population PDX supplementation was one of the factors significantly reducing triglyceride response compared to the placebo treatment (p < 0.05).

**Conclusions:**

Subjects with elevated fasting triglyceride levels display exaggerated and prolonged postprandial triglyceride responses. PDX, a soluble non-digestible carbohydrate, may offer a dietary concept for reducing the postprandial triglyceride response after the consumption of a meal containing a high concentration of fat.

## Background

Triglycerides and fatty acids are well known biomarkers of lipidemia. Although a definition of postprandial lipidemia (PPL) is yet to be widely agreed and adopted, PPL is generally accepted as a syndrome characterized by non-fasting hypertriglyceridemia associated with an increased risk of vascular events [1,2]. Furthermore, several epidemiological studies have determined that the delayed removal of postprandial triglyceride from the circulatory system is associated with atherosclerosis [3]. Patsch et al. [4] demonstrated that any concentration of postprandial triglyceride that remains in the system presents an independent risk factor for coronary artery disease. Indeed fasting triglyceride levels in overweight subjects are thought to be a reliable indicator of the risk of cardiovascular disease (CVD) [5]. The entrapment of chylomicron remnants within the subendothelial space and the concurrent stimulation of inflammatory reactions are processes that have both been presented as mechanisms which give rise to an increase in the risk of CVD due to increased levels of circulatory triglyceride [6]. PPL is also linked to increased oxidative stress, a contributor to endothelial dysfunction [7].

The effects of dietary, physiological, genetic and pathological influences on postprandial lipid metabolism have been reviewed by Lopez-Miranda et al. [3]. Insulin is an important regulator of lipid metabolism and so insulin resistance within the peripheral tissues is a link between metabolic syndrome and dyslipidemia [8]. Impaired postprandial triglyceride clearance is typically associated with the accumulation of visceral adipose tissue [9,10], increased waist circumference [11], obesity [5,12], metabolic syndrome [13], type 2 diabetes [14] as well as elevated levels of triglyceride during the fasting state [9,15]. In addition, various physiological factors and lifestyle conditions are known to increase PPL, such as age [16], male gender [17,18], some gene variants [3], menopause [19], use of alcohol [20] and a low level of physical activity [21]. Females demonstrate a less intense postprandial lipid response than men due to their increased clearance capacity enabling them to remove triglycerides from their circulatory system more quickly. Tolerance of fatty meals typically decreases with age but it may also be associated with weight gain or the menopausal phase. Conversely, increased physical exercise such as running or cycling, or even walking, can significantly reduce fasting triglyceride levels and the postprandial triglyceride response [22].

The main nutritional factor influencing postprandial lipidemia is the amount of fat present in a meal. Other components such as the presence of digestible carbohydrates, fibers and alcohol can mediate an effect but the details are as yet unclear [3]. The role of digestible and indigestible carbohydrates on postprandial lipid metabolism is reviewed by Lairon et al. [23] and the involvement of dietary fiber in the prevention of metabolic syndrome components by Galisteo et al. [24]. Although an increased intake of certain dietary fibers demonstrated a tendency to decrease postprandial lipid response [25], a controversy still exists surrounding the role of different dietary fibers on lipid response [26]. Generally, there are indications that various soluble fibers reduce the levels of fasting LDL without affecting the concentration of fasting triglyceride [27]. A reduced postprandial lipolysis due to the concomitant ingestion of fibers in a meal can be explained by various mechanisms. For instance, fibers that form viscous solutions, those that generate aggregates which contain lipids, and those that directly inhibit lipase activity [23] will all impact on the rate of lipolysis. Studies have shown that a diet enriched with oat bran is able to reduce fat absorption demonstrated by a measured increase in faecal fat associated with such diets [28].

Polydextrose (PDX), a non-digestible carbohydrate was used to test the efficacy of this fiber to reduce the triglyceride response. PDX is a highly branched, randomly bonded glucose polymer [29]. Due to its complex structure and profile of glycosidic bonds mammalian digestive enzymes are unable to digest PDX. Instead it is slowly fermented during its passage through the colon by colonic microbes producing short-chain fatty acids (SCFAs) [30,31]. PDX is a well-tolerated [32], low-caloric [29] fiber that can be easily incorporated into various food applications to reduce energy content and replace sugar and fat [33]. It has been recognized as soluble fiber, and human clinical, animal and *in vitro* studies have demonstrated several physiological effects associated with these features [34]. There are also indications that PDX can increase fasting HDL [35] and decrease postprandial triglyceride [36] concentrations.

Due to the importance of non-fasting triglyceride as a risk marker for cardiovascular diseases there is a need to understand the role of different nutrients which affect triglyceride metabolic regulation. However, there are very few studies which directly compare subjects with different lipid metabolism. This study examines postprandial lipid responses in normolipidemic, obese and hyperlipidemic subjects. The postprandial challenge model used in this study may provide a practical tool to test the efficacy of different nutrients to lower the triglyceride response.

## Methods

### Participants

The study was conducted in three research centers located in southern and central Finland. The study protocol of the normolipidemic subjects was approved by the Sports Institute of Finland (4.12.2004/Polydextrose study), the protocol for obese subjects by the Research Ethics Committee from the Hospital District of Northern Savo, Finland (2007/123) and the hyperlipidemic protocol by the Ethical Committee, Intermunicipal Hospital District of Southwest Finland (29.8.2006/346). The study was conducted according to the guidelines laid down in the Declaration of Helsinki. The purpose of the study was explained to participants who gave their written informed consent before being included in the study.

This study consists of three study populations: normolipidemic young adults, mildly hyperlipidemic overweight adults and obese non-diabetic adults. The main inclusion criteria for the normolipidemic subjects were total cholesterol < 5.0 mmol/l, triglyceride < 1.5 mmol/l and BMI < 30 kg/m^2^. The main inclusion criteria for the hyperlipidemic overweight subjects were fasting triglyceride 1.5-2.5 mmol/l. The main inclusion criteria for the obese non-diabetic subjects were BMI 30–37 kg/m^2^. Exclusion criteria for all the groups were: use of lipid lowering medication, antiobesity drugs, dietary supplements with high fiber content, pregnancy, cardiovascular conditions and metabolic diseases. A structured interview on previous and current diseases, current use of medication and alcohol and tobacco consumption was carried out during a screening visit to clarify the health status of the subjects and their suitability for the study. In addition, body weight and height were measured and fasting blood samples were taken at this time.

### Study design

All the studies were conducted as randomized, double-blind placebo-controlled, cross-over trials. Each study consisted of two periods (postprandial interventions) and a wash-out period of approximately 10 days between interventions. A standard high fat, hamburger meal was used as a postprandial lipidemia model as described by Ahotupa et al. [37].

Body weight and height were determined during the screening visit. All subjects were requested to keep their lifestyles and body weight consistent during the study. Subjects were also advised to avoid strenuous exercise and not to drink alcohol for 24 h before the test days and to avoid fat-rich foods on the day prior to the trial. Before the first postprandial test day subjects recorded everything they ate after 3 p.m. and they were encouraged to eat similarly during the day before the second postprandial test day. This was to standardize the diet on the day before the postprandial study. Use of nicotine containing products (maximal use: 10 cigarettes or equivalent daily) were noted from 24 hours after study commenced.

The normolipidemic and hyperlipidemic groups of subjects arrived at the trial center the morning after an overnight fast of 10–12 hours. Three hours before the high-fat test meal they were fed a light breakfast containing 1 sandwich with ham and cheese, and a glass of juice (total 2.9 g fat, 738 kJ). The obese group of subjects did not receive breakfast. Instead they were fed a high-fat test meal immediately after their overnight fast. The difference between the fasting and non-fasting baseline will be discussed later. All subjects ate a high-fat test meal (4293 kJ, 36% from fat). A drink was also provided which may or may not have included the test product, i.e. it was administered in a randomized order. The intervention meals were served at 10 a.m. and the last blood samples were taken at 4.30 p.m. Participants were given 20 minutes to consume the test meal and they were not allowed to consume any additional food during the 6-hour testing period, only drinking water was permitted. The subjects spent the trial days in the laboratory sitting and reading and all physical exercise was forbidden. Venous blood samples were collected twice before and five times after the study meal.

### Composition of the study meal

The study meal consisted of a standard hamburger, french-fries and carbonated drink. The energy and nutrient content of the study meal is presented in Table 1. The experimental meals contained either 12.5 g (normolipidemic group) or 15 g (hyperlipidemic and obese groups) of PDX (Litesse® Ultra™, DuPont) added to the carbonated drink (400 mL). The drink without PDX acted as the placebo. The PDX dose was lower for the (lighter) normolipidemic subjects than obese and hyperlipidemic subjects, however the amount of PDX per kg of weight across all three subject groups was equal (p > 0.05). The addition of PDX (4 kJ/g) to the drink increased the energy content of the meal compared to the placebo meal by 50 kJ in the normolipidemic group and 60 kJ in the obese and hyperlipidemic groups. A blinded sensory evaluation ensured that PDX did not change the appearance or taste of the drink. Two hundred mL of water was served to the participants two and four hours after the meal.

**Table 1:** **The energy and nutrient content of the study meal** ^*^

	Weight (g)/Volume (ml)	Energy (kJ)	Protein (g)	Carbohydrates (g)	Fat (g)	Fiber (g)	Salt (g)
Hamburger	219	2071	27	40 (incl. 8 g sugar)	25 (SFA 10 g)	3	2.3
French Fries	114	1423	5	42 (incl. 1 g sugar)	17 (SFA 3 g)	4	0.4
Carbonated drink	450	799	<0.1	42	<0.1	0	0
Total	783	4293	32	124	42	7	2.7

SFA, saturated fatty acid.

^*^Fineli® -Finnish Food Composition Database [http://www.fineli.fi/]).

### Blood sampling and analysis

Fasting blood samples were taken during the initial screening and used to analyze lipid profile, plasma glucose and serum insulin concentrations. The venous blood samples used for the triglyceride analysis were taken twice before the meal (0-sample), and 30, 60, 120, 240 and 360 min after the meal at meal during both postprandial intervention periods. The time interval between the 0-samples was at minimum 10 minutes and the presumed reliable fasting value was the mean of those two measurements. The plasma was separated by centrifugation (3000 rpm, 10–15 min) after which it was stored at −70 C until analysed.

Total plasma-cholesterol levels were analysed using the enzymatic - photometric method, the levels of HDL were analysed by direct measurement and the triglyceride concentrations were analysed by enzymatic - colorimetric assay using an automatic analyser (Roche/Hitachi MODULAR ANALYTICS, Roche Diagnostics GmbH, D-68298 Mannheim, Germany) and commercial reagents (Cholesterol CHOD-PAP Cat. NO. 11875540, HDL-Cholesterol CAT. No. 04713214, LDL-Cholesterol Cat. No. 03038777, Triglycerides GPO-PAP Cat. No. 11730711, Roche Diagnostics GmbH, Mannheim, Germany). The plasma-LDL concentrations were calculated using the Friedewald formula. Plasma-glucose concentrations were analysed by the hexokinase method using citrate-fluoride plasma and serum insulin concentrations to create an immunoluminometric assay which was evaluated using an Immulite 2000 Analyzer (Thermo Fisher Scientific Inc.).

### Statistical analyses

The triglyceride response data were modeled using a linear mixed effects model with fixed effects for treatment, time point, and their interaction well as covariates for baseline, gender, age and BMI. The model also had nested random effects according to the subgroup structure of the data: intercept terms for study and subject within study. Prior to modeling, the data were transformed using logarithmic transform in order to obtain good model fit to data.

The 6-hour incremental area under curve (iAUC; ignoring the area below fasting level), peak concentration (C_max_), and time to reach peak concentration (T_max_) comparisons were performed either using one-way ANOVA followed by pairwise comparisons using Tukey’s HSD test or Kruskal-Wallis test followed by pairwise comparisons using Mann–Whitney U test and Holm-Bonferroni p-value correction.

All the statistics calculations were performed using R software (R Core Team, R Foundation for Statistical Computing, version 3.1.2 [38] and nlme library, version 3.1-118, [39].

## Results

### Characteristics of the study participants

Twenty eight males and thirty females participated in the studies. The obese group experienced one subject drop-out due to personal reasons. All other subjects successfully completed the combination of two postprandial test days. The baseline characteristics of participants are summarized in Table 2. The hyperlipidemic group had more males than females (14/7) and in the obese group the ratio was vice versa (5/13). As expected, results for the normolipidemic group demonstrated that plasma glucose, triglyceride, total cholesterol and LDL concentrations were significantly lower and HDL higher compared to the obese and the hyperlipidemic groups. The mean age of the normolipidemic subjects (range 20–25 years) was significantly lower than in the other groups (range 21–58 years). On the other hand, obese subjects showed significantly lower plasma glucose and total cholesterol and also higher HDL concentrations than the hyperlipidemic subjects. Both obese and hyperlipidemic subjects were only mildly hyperlipidemic.

**Table 2:** **The demographic and clinical characteristics of the study participants**

Characteristic	Normolipidemic	Obese	Hyperlipidemic
Men/Women*	9/10	5/13	14/7
Age (years)	22.1 (20–25)	42.0 (26–53)***	42.0 (21–58)***
Weight (kg)	71.9 (60–88)	94.8 (73–114)***	88.6 (67–112)***
BMI (kg/m^2^)	24.1 (20–28)	33.6 (30–37)***	29.6 (21–37)***###
Glucose (mmol/L)	4.53 ± 0.52	5.65 ± 0.58***	6.66 ± 1.66***#
Insulin (mU/L)	10.26 ± 4.89	9.75 ± 6.81	11.29 ± 7.22
TG (mmol/L)	1.02 ± 0.32	1.71 ± 0.96**	1.62 ± 0.62***
Total cholesterol (mmol/L)	4.24 ± 0.84	5.28 ± 1.19**	6.32 ± 1.54***#
LDL-cholesterol (mmol/L)	2.45 ± 0.54	3.12 ± 0.96*	3.34 ± 0.83***
HDL-cholesterol (mmol/L)	2.33 ± 0.20	1.43 ± 0.51***	1.05 ± 0.36*#

Values are expressed as ratios*, mean values with their ranges (min-max) or standard deviations (±SD).

BMI, body mass index; TG, triglyceride; LDL, low density lipoprotein; HDL, high density lipoprotein.

Student’s t-test was used to test statistical differences between the study groups.

*p < 0.05, **p < 0.01, ***p < 0.001, compared to normolipidemic.

#p < 0.05, ###p < 0.001 compared to obese.

### Postprandial triglyceride responses in different study groups

**Figure 1:**
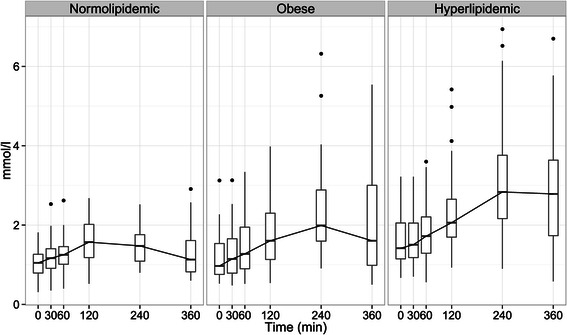
**Postprandial responses of plasma triglyceride to the high fat meal in different study groups at time points 30, 60, 120, 240, and 360 minutes.** Results are presented as box-plots, where the box represents the interquartile range, containing 50% of the values. The thin line inside the box shows the median of the values (which typically differs from the mean since the distributions of the values are often not symmetric). The whiskers extend from the box to the maximum and the minimum values. Outliers are plotted separately.

Postprandial changes in plasma triglyceride levels among different study groups are shown in Figure 1. The corresponding kinetic parameters: iAUC, C_max_ and T_max_ are shown in Table 3. TG C_max_ in normolipidemic group was significantly lower compared to hyperlipidemic (p < 0.001) or obese (p < 0.05) groups (1.8 ± 0.5, 2.48 ± 1.3 and 3.32 ± 1.47 mmol/l, respectively). TG C_max_ was also significantly higher in hyperlipidemic group compared to obese group (p < 0.01). Postprandial TG T_max_ occurred significantly earlier in normolipidemic (179 min) group compared to obese (252 min) (p < 0.01) or hyperlipidemic (271 min) (p < 0.001) groups. Normolipidemic subjects showed lower iAUC compared to the obese (p < 0.01) or hyperlipidemic (p < 0.001) subjects.

**Table 3:** **Kinetic parameters of plasma triglycerides**

Variable	Normolipidemic	Obese	Hyperlipidemic
iAUC (mmol/l × min)	135.0 ± 86	245.8 ± 147*	328.7 ± 190***##
C_max_ (mmol/l)	1.8 ± 0.5	2.48 ± 1.3*	3.32 ± 1.47***##
T_max_ (min)	179.1 ± 102	251.7 ± 72.9**	271.4 ± 70.4***

Values are shown as means ± standard deviations.

(iAUC), incremental area under curve; (C_max_), maximum concentration; (T_max_) time to achieve maximum concentration.

*p < 0.05, **p < 0.01, ***p < 0.001, compared to normolipidemic.

##p < 0.01 compared to obese.

**Figure 2:**
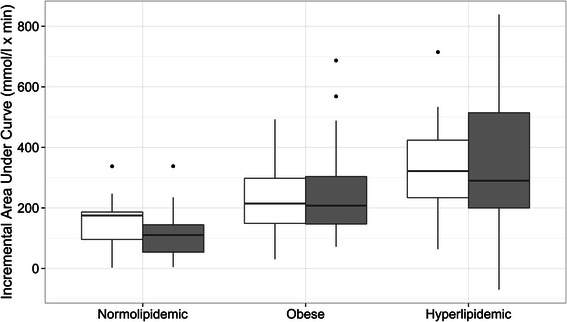
**The effect of placebo*(white)*and polydextrose (PDX)*(grey)*on the postprandial triglyceride response in normolipidemic, obese and hyperlipidemic groups.** The incremental areas under the curve (iAUC) for triglyceride responses in different study groups are presented as box-plots. The placebo and PDX treatment did not differ significantly in any of the study groups.

**Figure 3:**
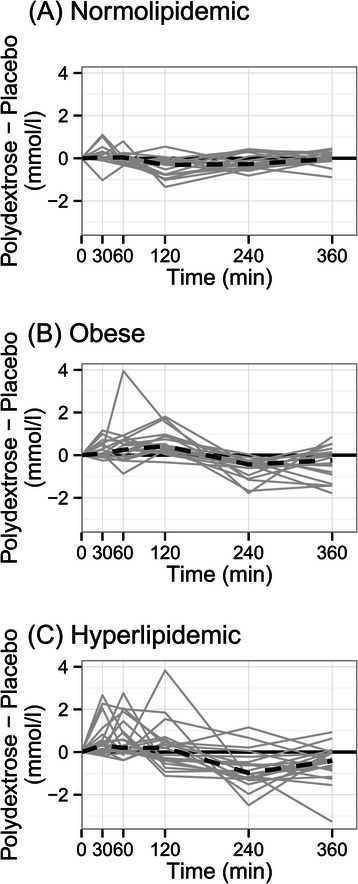
**The effect of polydextrose (PDX) on the individual postprandial triglyceride responses.** The curves represent the difference between the placebo response and PDX response for each individual in **A)** normolipidemic, **B)** obese, and **C)** hyperlipidemic groups. Solid lines (grey) show the individual responses and dashed line the median of the responses.

The effect of PDX on the iAUC in different study groups is shown in Figure 2. Due to the high biological variation of plasma triglyceride responses no significant differences in iAUC between placebo and PDX treatments were recorded. The effect of PDX on the individual triglyceride responses are shown in Figure 3. The curves represent the difference between the placebo response and PDX response for each individual.

### Factors affecting postprandial triglyceride responses

The baseline triglyceride value, PDX treatment, and time-point were all statistically significant (p < 0.05) factors explaining the differences in the postprandial triglyceride responses in the linear mixed-effects model. Irrespective of the study subpopulation, PDX supplementation resulted in lower values for the triglyceride response than the placebo treatment demonstrated by a statistically significant difference (p < 0.05). Notably the strongest decrease in response was observed in the normolipidemic group.

## Discussion

This study assessed triglyceride responses in subjects with both normal and compromised lipid types of metabolism. Results demonstrate that postprandial triglyceride responses were clearly elevated in those subjects with the highest baseline blood triglyceride concentrations. However, screening for hypertriglyceridemia which was based on fasting triglyceride levels is not able to highlight a definitive group of subjects with increased risk of excessive PPL. The variation in fasting triglyceride concentration was high amongst all the subjects and many the hyperlipidemic subpopulation exhibited only mild hyperlipidemia during the intervention (1.62 ± 0.62 mmol/l) despite being identified as hyperlipidemic according to the screenings data (1.5-2.5 mmol/l). Even though it was difficult to categorize hyperlipidemic subjects, the findings of this study are consistent with earlier findings which have shown that subjects with fasting hypertriglyceridemia display exaggerated and prolonged triglyceride responses [11,40]. In hyperlipidemic subjects the peak value was reached an average of 4 h 30 min after the test meal. In obese subjects this peak was reached slightly earlier (4 h 10 min), whereas for normolipidemic subjects the peak was reached by 3 h. The elevated triglyceride response in hyperlipidemic subjects that continued into the late postprandial phase (5–8 h) is associated with an increased risk of CVD [4].

Obesity is known to be an independent risk factor for cardiovascular diseases and it is linked to hyperlipidemia [41], however not all obese subjects in this study showed an excessive triglyceride response. Further, the statistical models showed that BMI did not significantly affect the triglyceride response. Generally, the magnitude of the triglyceride response of the obese group sits in between the normolipidemic and the hyperlipidemic groups. This emphasizes the importance to study the differences in metabolically healthy and abnormal obese subjects [42].

This study did not use the postprandial fat tolerance test to evaluate cardiovascular risk factors in patients [1] but focused on the typical meal induced triglyceride response in different target populations. However, the studies on the different target groups were performed in different years, environments and pre-breakfast status which presented some methodological limitations. More specifically, the normolipidemic subjects were mainly young, healthy subjects with a limited age range possibly explaining this group’s more limited variation in triglyceride response as compared to that for the hyperlipidemic and obese subjects. Moreover, studies with hyperlipidemic and obese subjects were bi-centric whereas normolipidemic study was single centric. Also, the unbalanced distribution of males to females in the obese and hyperlipidemic groups was a disadvantage to the study, but conversely the statistical analysis demonstrated that gender was not a factor that significantly affected triglyceride responses. Also, for this study the obese subjects were fasting in the true sense of its definition (10–12 h before the test meal) whereas the hyperlipidemic and normolipidemic subjects had a light breakfast 3 hours before the test meal. However, based on the study by Sundvall et al. [43] we propose that this light breakfast (2.9 g fat, 738 kJ) did not significantly alter the subsequent post-prandial effect caused by the fatty, high caloric test meal (42 g of fat and 4293 kJ). Although these methodological differences within the studies make it somewhat difficult to draw definite conclusions of the differences between the study groups this study provides information for a real-life postprandial lipid challenge among different populations. Moreover the effect of polydextrose was measured in a practical fiber dose and in a realistic meal context.

Although most epidemiological studies have shown that high-fasting triglyceride levels are a CVD risk factor [44,45], postprandial triglyceride responses have recently gained more attention as an independent risk factor for the development of atherosclerosis [46-48]. Hokanson and Austin [44] have determined that 1 mmol/L higher-fasting triglyceride is associated with a 14% increased risk of CVD after adjustment with other risk factors. The importance of addressing triglyceride responses, and dietary ways of reducing these responses, is underlined by the fact that approximately two thirds of cardiovascular events have been estimated to persist in spite of effective statin treatment [8]. Statins have been described as not as effective in lowering blood triglyceride as they are for lowering LDL.

Due to the large inter-person and daily variations in PPL the mixed-effects linear model was used to clarify the factors affecting the PPL response. The model considered the data as one population with nested substructure based on groups and individual subjects. It showed that PDX supplementation was one of the factors responsible for decreasing significantly PPL. However, the effect of PDX supplementation on PPL was not significant if the TG response was analysed only within the study groups (Figure 2).

Other proposed means for decreasing postprandial triglyceride responses include adding n-3 fatty acids, certain types of proteins or adding carbohydrates to the diet [23,49,50]. The present study demonstrated that supplementing a high fat meal with PDX can decrease postprandial triglyceride concentrations. This effect was most pronounced in the study’s normolipidemic group. Previous studies have also shown that the addition of a dietary fiber, especially derived from oat bran, can reduce postprandial lipemic responses especially in normal-weight subjects [23,25,36]. Further, a study by Shimomura et al. [36] demonstrated that PDX in combination with lactitol and calcium was shown to reduce the rate of increase in plasma triglyceride concentrations after the ingestion of chocolate. As well as dietary fiber and other dietary components, there are further factors that affect postprandial lipidemia, such as exercise and smoking [3]. For this study we limited the impact of these further factors on triglyceride responses. For example exercise habits [21] were controlled the day before the study and alcohol consumption was prohibited due to its negative effects on postprandial lipidemia [20], whilst in moderation it may have beneficial effects on low grade inflammatory conditions.

Regulation of lipid metabolism by dietary fibers is typically thought to be associated with the physicochemical properties of soluble fibers, such as viscosity [51]. However, this alone does not explain the different outcomes of the postprandial lipid studies with dietary fibers [26]. In this study, the low-viscous PDX reduced the postprandial triglyceride response induced by a high fat meal. The effect of PDX on the individual triglyceride responses in Figure 3 indicates that PDX has an effect on the kinetics of TG absorption. Although PDX seems to decrease the overall TG response it may even increase the TG absorption shortly after the meal. Although the mechanism is yet unclear, dietary fibers are known to modify lipid digestion and absorption [26]. In addition, the colonic fermentation of non-digestible carbohydrates and generation of subsequent metabolites may modulate lipid metabolism systemically or locally within the intestine. PDX is partially fermented in the colon and its fermentation metabolites have been shown *in vitro* to regulate the major genes (e.g. PPARα, PCG-1α, Lipin 1) involved with energy metabolism [52]. In a recent study by Olli et al. [53] PDX increased postprandial GLP-1 response which may partially explain the reduced triglyceride absorption of the PDX treatment.

## Conclusions

Postprandial triglyceride responses were most clearly exaggerated and prolonged in subjects with the highest baseline triglyceride concentrations. However, identifying the subjects with increased risk for excessive PPL is challenging due to the high intra-individual variation in the baseline triglyceride concentrations. The modelled triglyceride response of the combined study groups showed that in addition to the baseline triglyceride concentration also PDX treatment influenced the postprandial triglyceride response. Notably the decrease in postprandial TG with PDX supplementation was strongest in normolipidemic subjects. The decreased triglyceride response achieved by supplementing a high fat meal with PDX, may offer a practically relevant means of reducing atherosclerotic risk factors.

## Abbreviations

BMI: Body mass index
CAD: Coronary artery disease
C_max_: Peak concentration
CVD: Cardiovascular disease
HDL: High density lipoprotein
iAUC: Incremental area under the curve
LDL: Low density lipoprotein
PDX: Polydextrose
PPL: Postprandial lipaemia
SCFA: Short chain fatty acid
SD: Standard deviation
SFA: Saturated fatty acid
TC: Total cholesterol
TG: Triglyceride
T_max_: Time to reach peak concentration
T2D: Type 2 diabetes

## References

[1] Kolovou GD, Mikhailidis DP, Kovar J, Lairon D, Nordestgaard BG, Ooi TC (2011). Assessment and clinical relevance of non-fasting and postprandial triglycerides: an expert panel statement. Currt Vasc Pharmacol.

[2] Jackson KG, Poppitt SD, Minihane AM (2012). Postprandial lipemia and cardiovascular disease risk: Interrelationships between dietary, physiological and genetic determinants. Atherosclerosis.

[3] Lopez-Miranda J, Williams C, Lairon D (2007). Dietary, physiological, genetic and pathological influences on postprandial lipid metabolism. Br J Nutr.

[4] Patsch JR, Miesenböck G, Hopferwieser T, Mühlberger V, Knapp E, Dunn JK (1992). Relation of triglyceride metabolism and coronary artery disease. Studies in the postprandial state. Arterioscler Thromb.

[5] Hitze B, Rubin D, Helwig U, Schrezenmeir J, Bosy-Westphal A, Müller MJ (2008). Postprandial triglyceride response in men: role of overweight, abdominal fat and nutrition. Obes Facts.

[6] Klop B, Proctor SD, Mamo JC, Botham KM, Castro Cabezas M. Understanding postprandial inflammation and its relationship to lifestyle behaviour and metabolic diseases. Int J Vasc Med. 2012;2012:947417. 10.1155/2012/947417 PMC3179890 21961070

[7] Wallace JP, Padilla J, Mather K (2010). Postprandial lipaemia, oxidative stress and endothelial function: a review. Int J Clin Pract.

[8] Klop B, Elte JW, Castro CM (2013). Dyslipidemia in obesity: mechanisms and potential targets. Nutrients.

[9] Blackburn P, Lamarche B, Couillard C, Pascot A, Bergeron N, Prud’homme D (2003). Postprandial hyperlipidemia: another correlate of the “hypertriglyceridemic waist” phenotype in men. Atherosclerosis.

[10] Couillard C, Bergeron N, Prud’homme D, Bergeron J, Tremblay A, Bouchard C (1998). Postprandial triglyceride response in visceral obesity in men. Diabetes.

[11] Oka R, Kobayashi J, Miura K, Nagasawa S, Moriuchi T, Hifumi S (2009). Difference between fasting and nonfasting triglyceridemia; the influence of waist circumference. J Atheroscler Thromb.

[12] Lewis GF, O’Meara NM, Soltys PA, Blackman JD, Iverius PH, Druetzler AF (1990). Postprandial lipoprotein metabolism in normal and obese subjects: comparison after the vitamin A fat-loading test. J Clin Endocrinol Metab.

[13] Branchi A, Torri A, Berra C, Colombo E, Sommariva D. Changes in serum lipids and blood glucose in non diabetic patients with metabolic syndrome after mixed meals of different composition. J Nutr Metab. 2012;2012:215052. 10.1155/2012/215052 PMC3306966 22474578

[14] Iovine C, Vaccaro O, Gentile A, Romano G, Pisanti F, Riccardi G (2004). Post-prandial triglyceride profile in a population-based sample of Type 2 diabetic patients. Diabetologia.

[15] O’Meara NM, Lewis GF, Cabana VG, Iverius PH, Getz GS, Polonsky KS (1992). Role of basal triglyceride and high density lipoprotein in determination of postprandial lipid and lipoprotein responses. J Clin Endocrinol Metab.

[16] Cohn JS, McNamara JR, Cohn SD, Ordovas JM, Schaefer EJ (1998). Postprandial plasma lipoprotein changes in human subjects of different ages. J Lipid Res.

[17] Halkes CJ, Castro Cabezas M, van Wijk JP, Erkelens DW (2001). Gender differences in diurnal triglyceridemia in lean and overweight subjects. Intl J Obes Relat Metab Disord.

[18] Kashyap ML, Barnhart RL, Srivastava LS, Perisutti G, Allen C, Hogg E (1983). Alimentary lipemia: plasma high-density lipoproteins and apolipoproteins CII and CIII in healthy subjects. Am J Clin Nutr.

[19] van Beek AP, de Ruijter-Heijstek FC, Erkelens DW, de Bruin TW (1999). Menopause is associated with reduced protection from postprandial lipemia. Arterioscler Thromb Vasc Biol.

[20] Suter PM, Gerritsen-Zehnder M, Häsler E, Gürtler M, Vetter W, Hänseler E (2001). Effect of alcohol on postprandial lipemia with and without preprandial exercise. J Am College Nutr.

[21] Hardman AE, Lawrence JEM, Herd SL (1998). Postprandial lipemia in endurance-trained people during a short interruption to training. J ApplPhysiol.

[22] Peddie MC, Rehrer NJ, Perry TL (2012). Physical activity and postprandial lipidemia: are energy expenditure and lipoprotein lipase activity the real modulators of the positive effect?. Prog Lipid Res.

[23] Lairon D, Play B, Jourdheuil-Rahmani D (2007). Digestible and indigestible carbohydrates: interactions with postprandial lipid metabolism. J Nutr Biochem.

[24] Galisteo M, Duarte J, Zarzuelo A (2008). Effects of dietary fibers on disturbances clustered in the metabolic syndrome. J Nutr Biochem.

[25] Cara L, Dubois C, Borel P, Armand M, Senft M, Portugal H (1992). Effects of oat bran, rice bran, wheat fiber, and wheat germ on postprandial lipemia in healthy adults. Am J Clinl Nutr.

[26] Ulmius M, Johansson A, Önning G (2009). The influence of dietary fibre source and gender on the postprandial glucose and lipid response in healthy subjects. Eur J Nutr.

[27] Brown L, Rosner B, Willett WW, Sacks FM (1999). Cholesterol-lowering effects of dietary fiber: a meta-analysis. Am J Clin Nutr.

[28] Lia A, Andersson H, Mekki N, Juhel C, Senft M, Lairon D (1997). Postprandial lipemia in relation to sterol and fat excretion in ileostomy subjects given oat-bran and wheat test meals. Am J Clin Nutr.

[293] Auerbach MH, Craig SA, Howlett JF, Hayes KC (2007). Caloric availability of polydextrose. Nutr Rev.

[30] Fava F, Mäkivuokko H, Siljander-Rasi H, Putaala H, Tiihonen K, Stowell J (2007). Effect of polydextrose on intestinal microbes and immune functions in pigs. Br J Nutr.

[31] Probert HM, Apajalahti JH, Rautonen N, Stowell J, Gibson GR (2004). Polydextrose, lactitol, and fructo-oligosaccharide fermentation by colonic bacteria in a three-stage continuous culture system. Appl Environ Microbiol.

[32] Flood MT, Auerbach MH, Craig SA (2004). A review of the clinical toleration studies of polydextrose in food. Food Chem Toxicol.

[33] Murphy O (2001). Non-polyol low-digestible carbohydrates: food applications and functional benefits. Br J Nutr.

[34] Raninen K, Lappi J, Mykkanen H, Poutanen K (2011). Dietary fiber type reflects physiological functionality: comparison of grain fiber, inulin, and polydextrose. Nutr Rev.

[35] Schwab U, Louheranta A, Torronen A, Uusitupa M (2006). Impact of sugar beet pectin and polydextrose on fasting and postprandial glycemia and fasting concentrations of serum total and lipoprotein lipids in middle-aged subjects with abnormal glucose metabolism. Eur J Clin Nutr.

[36] Shimomura Y, Maeda K, Nagasaki M, Matsuo Y, Murakami T, Bajotto G (2005). Attenuated response of the serum triglyceride concentration to ingestion of a chocolate containing polydextrose and lactitol in place of sugar. Biosci Biotechnol Biochem.

[37] Ahotupa M, Suomela JP, Vuorimaa T, Vasankari T (2010). Lipoprotein-specific transport of circulating lipid peroxides. Ann Med.

[38] R Core Team. R Foundation for Statistical Computing, version 3.1.2. [http://www.R-project.org/]

[39] Pinheiro J, Bates D, Saikat D, Deepayan S, R Core Team. nlme: Linear and Nonlinear Mixed Effects Models, version 3.1-118, [http://CRAN.R-project.org/package=nlme]

[40] Alcala-Diaz JF, Delgado-Lista J, Perez-Martinez P, Garcia-Rios A, Marin C, Quintana-Navarro GM (2014). Hypertriglyceridemia influences the degree of postprandial lipemic response in patients with metabolic syndrome and coronary artery disease: from the CORDIOPREV study. PLoS One.

[41] Lakka HM, Lakka TA, Tuomilehto J, Salonen JT (2002). Abdominal obesity is associated with increased risk of acute coronary events in men. Eur Heart J.

[42] Perez Martinez P, Alcala Diaz JF, Delgado Lista J, Garcia Rios A, Gomez Delgado F, Marin Hinojosa C (2014). Metabolic phenotypes of obesity influence triglyceride and inflammation homoeostasis. Eur J Cin Nutr.

[43] Sundvall J, Laatikainen T, Hakala S, Leiviskä J, Alfthan G (2008). Systematic error of serum triglyceride measurements during three decades and the effect of fasting on serum triglycerides in population studies. Clin Chim Acta.

[44] Hokanson JE, Austin MA (1996). Plasma triglyceride level is a risk factor for cardiovascular disease independent of high-density lipoprotein cholesterol level: a meta-analysis of population-based prospective studies. J Cardiovasc Risk.

[45] Sarwar N, Danesh J, Eiriksdottir G, Sigurdsson G, Wareham N, Bingham S (2007). Triglycerides and the risk of coronary heart disease: 10,158 incident cases among 262,525 participants in 29 Western prospective studies. Circulation.

[46] Adiels M, Matikainen N, Westerbacka J, Söderlund S, Larsson T, Olofsson SO (2012). Postprandial accumulation of chylomicrons and chylomicron remnants is determined by the clearance capacity. Atherosclerosis.

[47] Bansal S, Buring JE, Rifai N, Mora S, Sacks FM, Ridker PM (2007). Fasting compared with nonfasting triglycerides and risk of cardiovascular events in women. JAMA.

[48] Nordestgaard BG, Benn M, Schnohr P, Tybjærg-Hansen A (2007). Nonfasting triglycerides and risk of myocardial infarction, ischemic heart disease, and death in men and women. JAMA.

[49] Williams CM, Moore F, Morgan L, Wright J (1992). Effects of n-3 fatty acids on postprandial triacylglycerol and hormone concentrations in normal subjects. Br J Nutr.

[50] Higashi K, Abata S, Iwamoto N, Ogura M, Yamashita O, Ishikawa O (2001). Effects of soy protein on levels of remnant-like particles cholesterol and vitamin E in healthy men. J Nutr Sci Vitaminol.

[51] Dikeman CL, Fahey GC (2006). Viscosity as related to dietary fiber: a review. Crit Rev Food Sci Nutr.

[52] Putaala H, Makivuokko H, Tiihonen K, Rautonen N (2011). Simulated colon fiber metabolome regulates genes involved in cell cycle, apoptosis, and energy metabolism in human colon cancer cells. Mol Cell Biochem.

[53] Olli K1, Salli K, Alhoniemi E, Saarinen M, Ibarra A, Vasankari T (2015). Postprandial effects of polydextrose on satiety hormone responses and subjective feelings of appetite in obese participants. Nutr J.

